# Nuclear bomb and public health

**DOI:** 10.1057/s41271-023-00420-x

**Published:** 2023-06-14

**Authors:** Shan Xu, Alicia Dodt

**Affiliations:** 1 University Hospital Essen, Essen, Germany; 2 Department of Oncology, Mianyang Central Hospital, MianYang, China; 3 Hochschule Niederrhein University of Applied Sciences, Krefeld, Germany

**Keywords:** Atomic bomb, Nuclear bomb, Nuclear weapons, Rescue strategies

## Abstract

Since the nuclear bomb attack against Hiroshima and Nagasaki in 1945, the world has advanced in nuclear technology. Today, a nuclear bomb could target a large-scale attack, at a longer range, and with much greater destructive force. People are increasingly concerned about the potential destructive humanitarian outcomes. We discuss actual conditions detonation of an atomic bomb would create, radiation injuries, and diseases. We also address concerns about functionality of medical care systems and other systems that support medical systems (i.e., transport, energy, supply chain, etc. systems) following a massive nuclear attack and whether citizens able to survive this.

## Key messages

- So long as nuclear weapons exist, it is inevitable that someday they will be used, whether by design, accident, or miscalculation.
- We address what would happen during a nuclear bomb explosion in a city and whether citizens able to survive this.

## Introduction

**Fig. 1:**
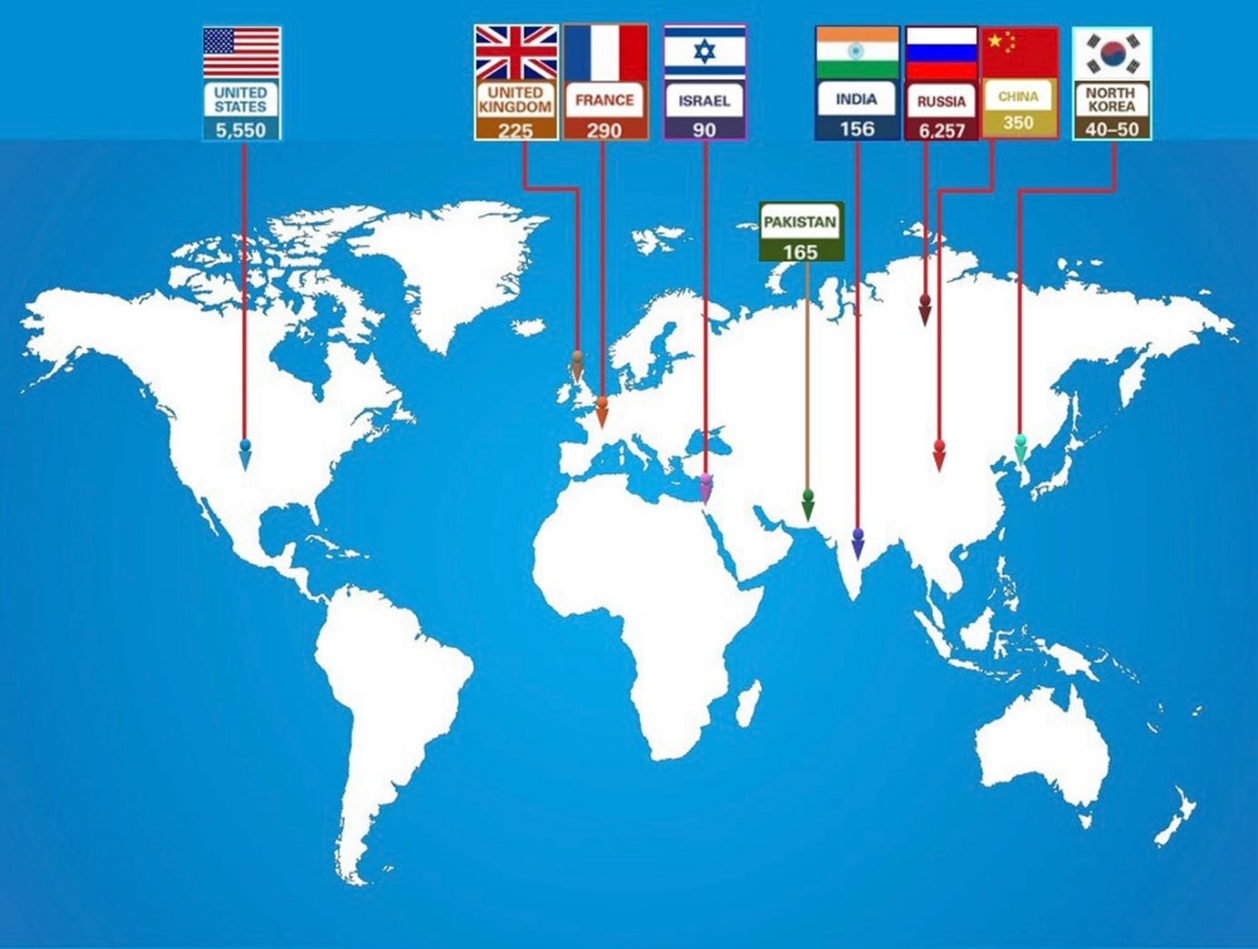
The estimated global nuclear warhead inventories

The end of World War II heralded the atomic age, and many countries embarked on a nuclear arms race [1]. From 1945 to 1964, the United States, the Union of Soviet Socialist Republics (USSR), the United Kingdom, France, and China successively became nuclear-armed countries [2, 3]. By early 2022, nine countries possessed a total of approximately 13,000 warheads[4] (Fig. 1). The danger of use of nuclear weapons is greater than ever before due to proliferation of nuclear weapons, terrorism, and political instabilities [5]. Russia's recent invasion of Ukraine has heightened the nuclear war risks thus the public is increasingly concerned about nuclear weapons [6–8]. No researchers have recently analyzed the impact of a nuclear detonation and diagrams presented in newspapers are not useful [9].

This Viewpoint addresses what would happen during a nuclear bomb explosion in a city and whether citizens are able to survive. We discussed a scenario of a single 550 kilotons (KT) bomb detonated on a city, as the most common size of strategic nuclear bomb in the Russian arsenal is a 550 KT yield [10].

## A nuclear bomb explosion in a city

### A nuclear bomb detonated in the air

A nuclear explosion first causes an intense light flash to appear, which comes with a strong pulse of thermal radiation [11]. The radiation is sufficiently powerful to ignite light combustibles as far away as 14 km[12]. A strong pulse of gamma-rays appears with a lethal scope of 3 km, thus the subsequent blast would kill almost all individuals within that scope. A blindingly bright flash follows, with a ‘fire ball’ rising for a few seconds that radiates immense heat [13]. On a day or night without clouds, individuals within a scope of 8 km would suffer from permanent or transient blindness. All exposed body parts would experience deep burning into the flesh within 10 km and high susceptibility to superficial burning would reach 15 km or more [13]. The initial flash would ignite many fires, so the scorched clothing would cause burns. Extra fuel from collapsed buildings and burst fuel tanks and gas mains would likely combine into a ‘firestorm’. A fire wind resulting from an updraft of coalescing fires blown inwards from all directions at gale force would intensify the fire. These conditions would hinder even the uninjured as they try to run or walk out of the fire. Temperatures in bomb shelters and basements would rise above fatal levels, and the fire would completely consume available oxygen [12].

**Fig. 2:**
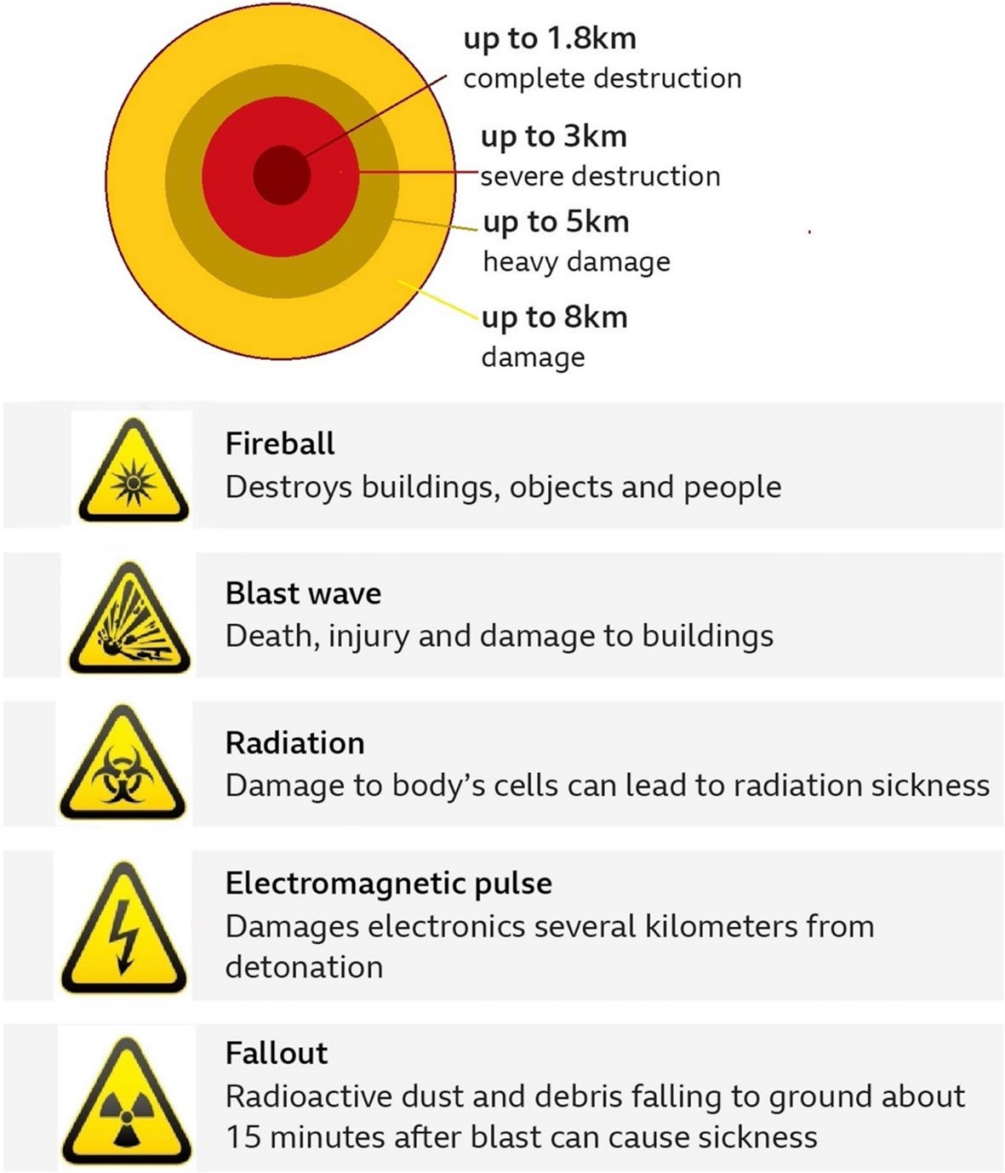
An estimate of the size of the damage caused by an atomic bomb

A tremendous power wave, commencing with the light flash, propagates at slower velocities. Its destructive radius of 2 km for reinforced concrete structures would reach as far as 8 km for timber or brick frame structures. Houses within a scope of 14 km would undergo major damage, and windows at distances of 20–30 km could break [13]. Prior to the blast strike, individuals sufficiently far away would have several seconds to lie down or dive into a hollow or ditch. Nearly all individuals within 3 km would be killed by the direct blast or by collapsing or flying masonry. Approximately 50% of individuals within 8 km would be killed by the blast [13]. Winds at the force of a hurricane follow the blast wave. Initially, these blow outwards from the explosion; after seconds, they blow inwards to replenish the air. Within 4 km, the wind could have a velocity of 600 km/hr and the force of a tornado, meaning it could drive glass splinters, and knock down wooden utility poles (Fig. 2).

### A nuclear bomb detonated on the ground

A ground-level explosion would create a gigantic crater ––a major difference from an explosion in the urban sky [12]. Rocks, dirt, and masonry would turn into radioactive dust and debris. Larger particulates would settle rapidly and finer ones more slowly, primarily downwind from the explosion [13]. An explosion at a harbor would also create a crater –and a tidal wave. Most harbor water would turn radioactive, spray into the sky, and cause radioactive downpour [12]. Thus, severe radioactive fallout would probably render the city, along with affected downwind regions up to 10 km, uninhabitable for years [14]. Compared to an airburst, the blast would probably cause damage to an area half the size. Extra structural damage would affect buildings as if there had been an earthquake. Although immediate deaths would probably number half those from an airburst, many would die of radiation sickness, especially survivors who had no shelter from fallout.

## Ionizing radiation from a nuclear bomb

**Fig. 3:**
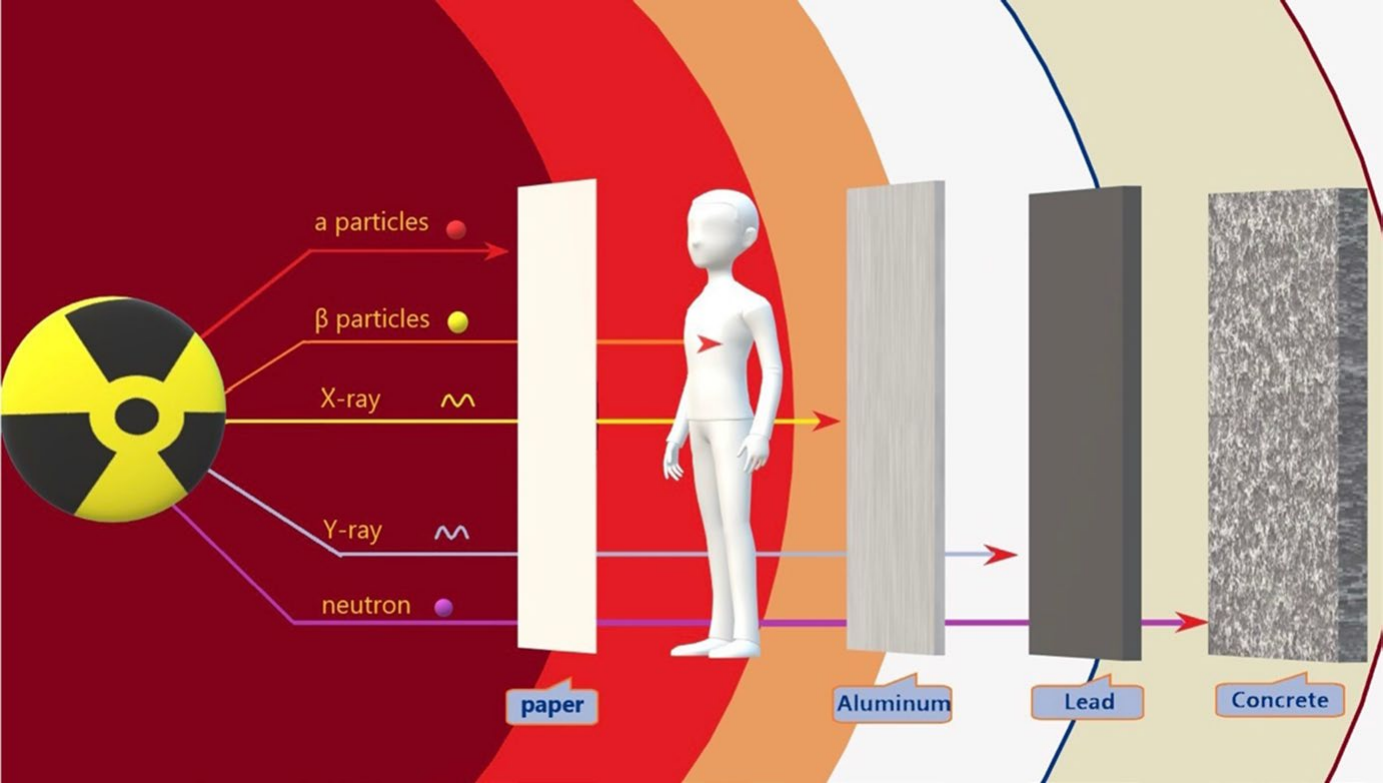
Type of ionizing radiation

**Fig. 4:**
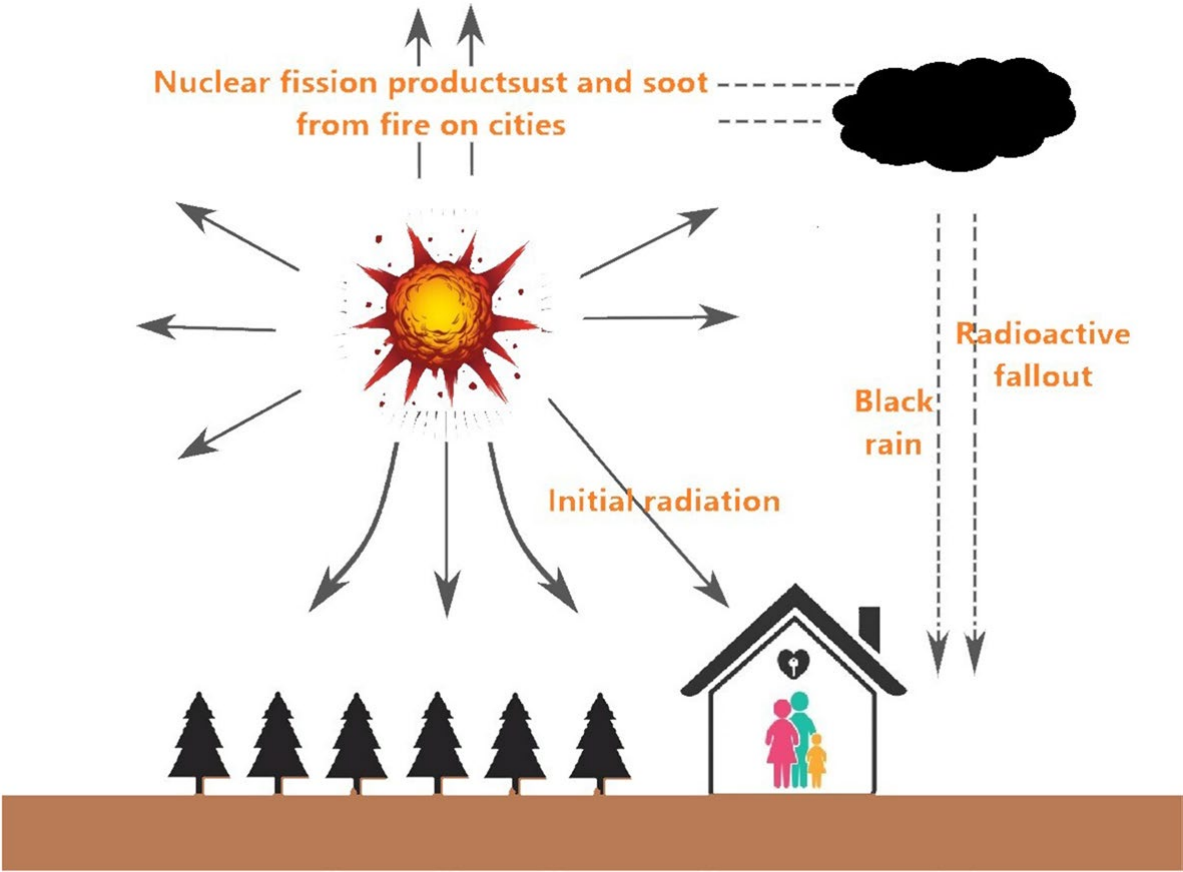
The ionizing radiation released from atomic bombs

Ionizing radiation release refers to a unique phenomenon in nuclear explosions [15]. It is a type of energy released by atoms in the form of electromagnetic waves or particles that could travel unseen and pass through materials (Fig. 3). Nuclear air bursts form ionizing radiation, such as γ-rays and neutrons [16]. The ionizing activity can alter molecules within the cells of our bodies, causing cancer and death. Ionizing radiation from explosion of atomic bombs consists of initial and residual radiation [17] (Fig. 4). Initial radiation can result directly from nuclear fission and fission products of fireballs, causing exposure to over ground γ-rays and neutrons [18]. An initial nuclear radiation level drops quickly based on the distance from a fireball; less than 1 roentgen reaches 8 km from ground zero [19]. The roentgen is a legacy unit to measure radiation exposure by a person over a period of time. Residual radiation results from neutron-caused radioactive materials in the environment as well as fission products in fallout (Delayed Radiation) [17]. As fission products cool after rising to high atmospheric layers, the fallout usually drops to ground after a nuclear bomb blast. This rain is a mixture of dust and soot from urban remains. More than 300 fission products may be generated from a fission reaction, and multiple fission products are radioactive with great differences in half-lives –from a second to months or years [12].

## Diseases and death associated with radiation

### Early symptoms of radiation

Researchers have observed major symptoms including epilation, petechiae, along with fever, diarrhea, vomiting, additional oropharyngeal diseases, and hemorrhagic manifestations [16]. Epilation is an important symptom. Patients who experience it typically show more involvement of the crown than bilateral sides [20, 21]. Petechiae, tiny round spots that appear on the skin as a result of bleeding, can be seen on extremities or pressure points. Great ecchymoses, dark purple spots on the skin where blood has leaked out of blood vessels, can be seen around needle puncture sites, with free bleeding in partly healed wounds. Retinal hemorrhage occurs in most cases, with extended bleeding and coagulation time. Platelet counts decrease dramatically. Japanese researchers reported nausea and vomiting starting several hours after an explosion, with improvement the next morning, but sometimes lasting for 2–3 days [22, 23].

### Long-term effects of radiation

Radiation exposure causes acute, near-immediate impact by killing cells and injuring tissues, and may generate additional illness, including cancer, through triggering mutations of DNA in live cells [24]. Clinicians believe that mutations are accumulated in specific cells and their progeny [25]. An increase in the incidence of radiation-caused cancer may be noted many years after exposure [26]. Among chronic impacts on atomic bomb survivors, the most lethal is leukemia [27]; research showed an increase two years after nuclear bomb that peaked after approximately 4 to 6 years [28]. Children were most seriously affected [29].

Kamiya K, Ozasa K et al.found incidence of other cancers increased approximately a decade after the attacks [30]. Preston et al.showed the attributable rate of radiation exposure to solid cancer was lower than that to leukemia by 10.7% [31]. Data from Radiation Effects Research Foundation (RERF) reveal that for an individual with exposure to an almost non-survivable whole-body radiation dose, the solid cancer risk is up to five times higher than that for an unexposed individual [32, 33]. Another concern is for children of these survivors. For those exposed to radiation prior to birth (in utero) research shows increased rates of children with small head size, mental disability, and impaired physical growth. Those exposed in utero had lower cancer rate increases than those exposed during childhood [30]. Approximately 40% of the A-bomb survivors of Hiroshima and Nagasaki are alive today [34]. After the bombing attacks in Hiroshima and Nagasaki, concern grew about the health effects of radiation on the future of children of survivors [34]. At present, no radiation-associated excess disease has been noted among children–but more time will be needed to verify these findings [35].

## Rescue serviced during a nuclear attack in a city

When a bombing occurs in a city center, rescue services there will suffer severe structural damage and may fail to work [36]. Hospitals in the damaged area will be destroyed, and no electricity, water, and telephone communication will be available [12]. Impaired areas will be inaccessible for external rescue services [37] or emergency services in nearby cities will be overwhelmed given the large number of injured individuals [12].

The destroyed city is radioactive. Rescue work should be conducted by specialized teams with proper protection, based on how much radiation the team could tolerate[38]. Planners should also consider the willingness for team members to bear the risk. Injured individuals sent to the hospitals must accept radioactivity testing to ensure staff safety. Such testing requires proper equipment and trained personnel and could cause severe delays. Even though timely therapy could have saved the exposed individuals, most of those injured will die. A few might be rescued, but most may die in hours or days given lack of analgesics, food, water, or other aids.

## Medical supply and resources after a nuclear attack

The Office of Technology Assessment (OTA) in the United States (U.S) depicted damage anticipated in Detroit, a select U.S city [39]. They assumed an evening attack with detonation at an altitude of 6000 feet, without warning, and with no other cities attacked. According to their calculation, 470,000 of the population of 4.3 million would die, 630,000 would be injured. Of the nuclear bomb survivors in Japan, 70% suffered blast injuries, 65% burns, and 20% radiation injuries [40]. This implies that 440,000 would have blast injuries, 409,000 burns injuries, and 157,000 would suffer moderate or severe radiation. A high proportion of severe burn injured patients would require hospitalization and special care. These injuries require specialized equipment, specially trained medical personnel and supplies. Burns of second or third degree covering 20% of the body surface can be fatal without weeks of intensive therapy in the hospital and long-term rehabilitation. Even with the use of current advanced medical techniques, mortality would be tremendous. Trauma poses similar problems. Thousands of patients would be unable to get timely care (bleeding prevention, administration of fluids, wound cleaning, and treatment of infections). Even if all the hospitals were to have intact medical personnel and supplies, it would not be possible to treat so many casualties quickly. Patients exposed to large doses of radiation need hospitalization.

The OTA’s Detroit estimate indicated the 630,000 patients would need 352,000 hospital beds, 42,000 burn beds, 134,000 intensive care beds, 13,333 physicians, 154,000 nurses, 4632 medical technologists, 8390 pharmacists and 3475 radiologic technologists [39], many more than available (1333 burn beds of 42, 000 needed, 61, 000 intensive care beds of 134, 000 needed). One doctor would care for 663 patients, one plastic surgeon 15,634 patients, and one nurse at least 154 patients. Blood products would fall far short of need: 12,000 units of platelets at hand of 15 million needed, 191,000 units of red blood cells of 1.28 million, and 57,000 units of whole blood of 1.28 million.

If 76% of the city's hospitals were to survive, other sorts of beds would be in short supply: 41 for burns of 42,000 needed, 1900 for intensive care of 134, 000, and 41,000 for hospitalization of 352,000 needed [39]. For blood and resuscitative fluids, 1800 units of whole blood would be available, but 1.28 million needed.

The nuclear weapon would generate a great electromagnetic pulse and destroy power sources and electronic equipment [41]. In the absence of power, it would be impossible to operate the life support instruments. Medical and rescue workers would confront huge problems apart from lacking care facilities: searching for the injured among the collapsed buildings and transporting them through ruined streets without vehicles.

## Self-rescue options after a nuclear attack and after being exposed to nuclear radiation

Such large-scale disasters would rapidly overwhelm even the most prepared city. Radiation challenges survival after explosion. A basement made of concrete would provide good protection, as would an underground parking lot, air-raid shelter, or subway station. If there is no basement, higher floors will be safer from radiation that will land on the ground. At least two floors above those sheltering this way would be needed to avoid radiation that settles on the roof. Restrooms and stairwell cores far from deposited radioactive fallout provide superior protection compared to those near roofs, windows, or exterior walls. Survivors can use plastic, tape, newspapers, or clothing to seal air gaps to prevent radioactive dust from entering. None of these measures are of use in case of a direct hit.

For those sheltering downwind or in areas several miles from ground zero, should stay in the basement for about three days [42]. Radiation is the strongest on the second day after the explosion, then drops (one-third on day 3, and one-fifth on day 5) [18]. Those upwind could stay at home if the fallout plume does not blow toward them. An emergency radio could be helpful to learn when rescue teams are nearby and when it is safe to go outside. Without clear guidance on the safety of leaving sooner, it is better to shelter for a week, and when leaving the shelter, survivors have to cover exposed skin and wear a N95/N100 mask to prevent inhalation of radiation as well as goggles to prevent radiation absorb through the eyes. These protections, however, are limited with no guarantee.

Given a person would know about own high radiation level, able to measure it frequently, and able to find safe settings with running water and supplies of soap, towels, clothing, and disposal facilities for radioactive material, the next steps are critical:

- Take off the outer layer of clothing (that may prevent exposure for up to ninety percent of radioactive material).
- Move gently to avoid shaking off radioactive dust.
- Clean oneself by warm shower, use soap and rinse.
- Cover cuts and abrasions when cleaning to prevent radioactive material from entering open wounds.
- Clean hands, face, and body using a sink or faucet (if no shower is possible) using soap and lots of water.
- Clean hands and face especially; use a moist wipe, clean wet cloth, or a damp paper towel (if there is no sink or faucet).
- Clean below the nose, their eyelids, eyelashes, and ears.
- Seal clothing, used wipes, cloth, or towels in a plastic bag away from people and pets.

Radioactive iodine will be released by nuclear blast; it can be inhaled or absorbed in food and water. Thus, exposed person should use potassium iodide (KI) to rinse the body to avoid accumulation in the thyroid gland and take a KI pill immediately after a nuclear blast to prevent the thyroid gland from absorbing radiated particles. Acute radiation syndromes, such as nausea, vomiting, and headaches, are associated with multiple severe systemic multi-organ failures and death [43]. Unfortunately, acute radiation syndromes require professional treatment in a hospital (stimulating hematopoiesis with growth factors, stem cell transfusions, or platelet transfusions) [44]. Antimicrobial agents (quinolones, ciprofloxacin, tetracyclines, antifungal agents, and fluoroquinolones) typically offer radioprotection [45]. Antiemetic agents (analgesic agents, antacids and H2 blockers) may help relieve symptoms. Radioprotectors, (amifostine, palifermin, genistein, and cordyceps sinensis) would also prevent radiation side effects [46–48].

## Conclusions

Nuclear weapons are the most dangerous weapons on earth. A single bomb can destroy a city, kill millions, and jeopardize the environments as well as future generations. Societies and individuals should prepare plans for such a disaster. The only effective way to deal with such danger is disarmament. Governments need to act to totally eliminate the nuclear weapons. Whole of societies must engage in pushing against a nuclear world, only then will governments be motivated to decrease the systemic risk.

## Data Availability

Not applicable.
