# Correction to: Nuclear bomb and public health

**DOI:** 10.1057/s41271-023-00429-2

**Published:** 2023-08-04

**Authors:** Shan Xu, Alicia Dodt

**Affiliations:** 1 University Hospital Essen, Essen, Germany; 2 Department of Oncology, Mianyang Central Hospital, MianYang, China; 3 Hochschule Niederrhein University of Applied Sciences, Krefeld, Germany

## Correction to: Journal of Public Health Policy (2023) 10.1057/s41271-023-00420-x

In this article, the first affiliation for Shan Xu should have been 'University Hospital Essen'.

The original article has been corrected.

